# Alcohol consumption, but not smoking is associated with higher MR-derived liver fat in an asymptomatic study population

**DOI:** 10.1371/journal.pone.0192448

**Published:** 2018-02-05

**Authors:** Christian Bayerl, Roberto Lorbeer, Margit Heier, Christa Meisinger, Susanne Rospleszcz, Anina Schafnitzel, Hannah Patscheider, Sigrid Auweter, Annette Peters, Birgit Ertl-Wagner, Maximilian Reiser, Fabian Bamberg, Holger Hetterich

**Affiliations:** 1 Institute of Clinical Radiology, Ludwig-Maximilian University Hospital, Munich, Germany; 2 Institute of Epidemiology II, Helmholtz Zentrum München, Neuherberg, Germany; 3 KORA Myocardial Infarction Registry, Central Hospital of Augsburg, Augsburg, Germany; 4 Ludwig-Maximilians-Universität München, UNIKA-T Augsburg, Germany; 5 German Center for Diabetes Research (DZD e.V.), Neuherberg, Germany; 6 German Center for Cardiovascular Disease Research (DZHK e.V.), Munich, Germany; 7 Department of Diagnostic and Interventional Radiology, Eberhard Karls University Tübingen, Tübingen, Germany; Medizinische Fakultat der RWTH Aachen, GERMANY

## Abstract

**Background:**

The aim of our study was to determine the relation of alcohol consumption and cigarette smoking on continuous-measured hepatic fat fraction (HFF) in a population free of cardiovascular disease. We suggested a direct correlation of alcohol consumption with HFF and increased HFF in former smokers compared to current smokers.

**Methods:**

Data from 384 subjects (mean age: 56 years, 58% men) of a population-based cohort study (KORA) were included in a cross-sectional design. Liver fat was assessed by 3 Tesla magnetic resonance imaging (MRI) using a multi-echo Dixon sequence and T2-corrected single voxel multi-echo spectroscopy (^1^H-MRS). Smoking status was classified as never, former or current smoker and alcohol consumption as non-, moderate (0.1–39.9 g/day for men and 0.1–19.9 g/day for women), or heavy drinker (≥ 40 g/day for men and ≥ 20 g/day for women). Fatty liver disease was defined as HFF≥5.56%.

**Results:**

Average HFF was 8.8% by ^1^H-MRS and 8.5% by MRI. Former smokers showed a higher HFF (MRI: β = 2.64; p = 0.006) and a higher FLD prevalence (MRI: OR = 1.91; p = 0.006) compared to never smokers. Current smokers showed decreased odds for FLD measured by ^1^H-MRS after multivariable adjustment (OR = 0.37; p = 0.007) with never smoker as reference. Heavy drinking was positively associated with HFF (^1^H-MRS: β = 2.99; p = 0.003) and showed highest odds for FLD (^1^H-MRS: OR = 3.05; p = 0.008) with non-drinker as reference. Moderate drinking showed a positive association with HFF (^1^H-MRS: β = 1.54; p = 0.061 and MRI: β = 1.75; p = 0.050).

**Conclusions:**

Our data revealed lowest odds for FLD in current smokers, moderate drinkers showing higher HFF than non-drinkers and heavy drinkers showing highest HFF and odds for FLD. These findings partly conflict with former literature and underline the importance of further studies to investigate the complex effects on liver metabolism.

## Introduction

Fatty liver disease (FLD) is a chronic disease characterized by increased accumulation of fat in hepatocytes as defined by imaging or histology [1]. While benign hepatic steatosis is completely reversible, FLD can progress to steatohepatitis and cirrhosis with potential detrimental complications like end stage liver disease and hepatocellular carcinoma [1, 2]. Hepatic fat deposition is triggered and influenced by numerous factors including medication, genetic predisposition, various systemic diseases and lifestyle [3, 4].

Although lifestyle factors including alcohol consumption and cigarette smoking are targeted in current prevention and treatment programs for FLD, literature data on the effects of both alcohol and smoking on FLD is conflicting:

It is well known that excessive alcohol consumption leads to persistent liver damage, which increases with the amount of alcohol consumed [5, 6]. However, several studies have revealed an inverse association between light to moderate alcohol consumption and the prevalence of FLD [7–11].

With regard to smoking, studies have suggested effects on the deposit and distribution of fat, mainly represented by BMI and waist-to-hip ratio [12–17]. For example, Dare et al. showed a lower risk for obesity in current smokers compared to never smokers; under smokers the risk for obesity increased with the amount of cigarettes smoked and former smokers had a higher risk for obesity compared to never and current smokers [18]. It could be assumed that associations between smoking and BMI may be similar to the associations between smoking and the deposition of fat in the liver, but former studies are controversial: some studies claim that there are no differences in FLD prevalence between current smokers and never smokers with no influence on the histological features or severity of FLD [19, 20], whereas others have shown increased prevalence of FLD in smokers [21, 22].

These studies, however, relied on ultrasonographic criteria and liver enzyme measurements for diagnosis of FLD, although ultrasound is known to be insensitive for low amounts of liver fat and does not allow for continuous and quantitative measurement of hepatic fat fraction [23, 24]. Recently, magnetic resonance imaging (MRI) and proton magnetic resonance spectroscopy (^1^H-MRS) have emerged as non-invasive reference standards for the quantitative assessment of liver fat with excellent correlation to histopathology [25].

The aim of our study was to investigate the association between cigarette smoking, alcohol consumption and HFF and FLD as defined by MRI and ^1^H-MRS in a population free of cardiovascular diseases. We hypothesized (i) that alcohol consumption is directly correlated with HFF and FLD prevalence, and (ii) that we find increased HFF and FLD prevalence in former smokers in comparison to current smokers.

## Methods

### Study population

**Fig 1:**
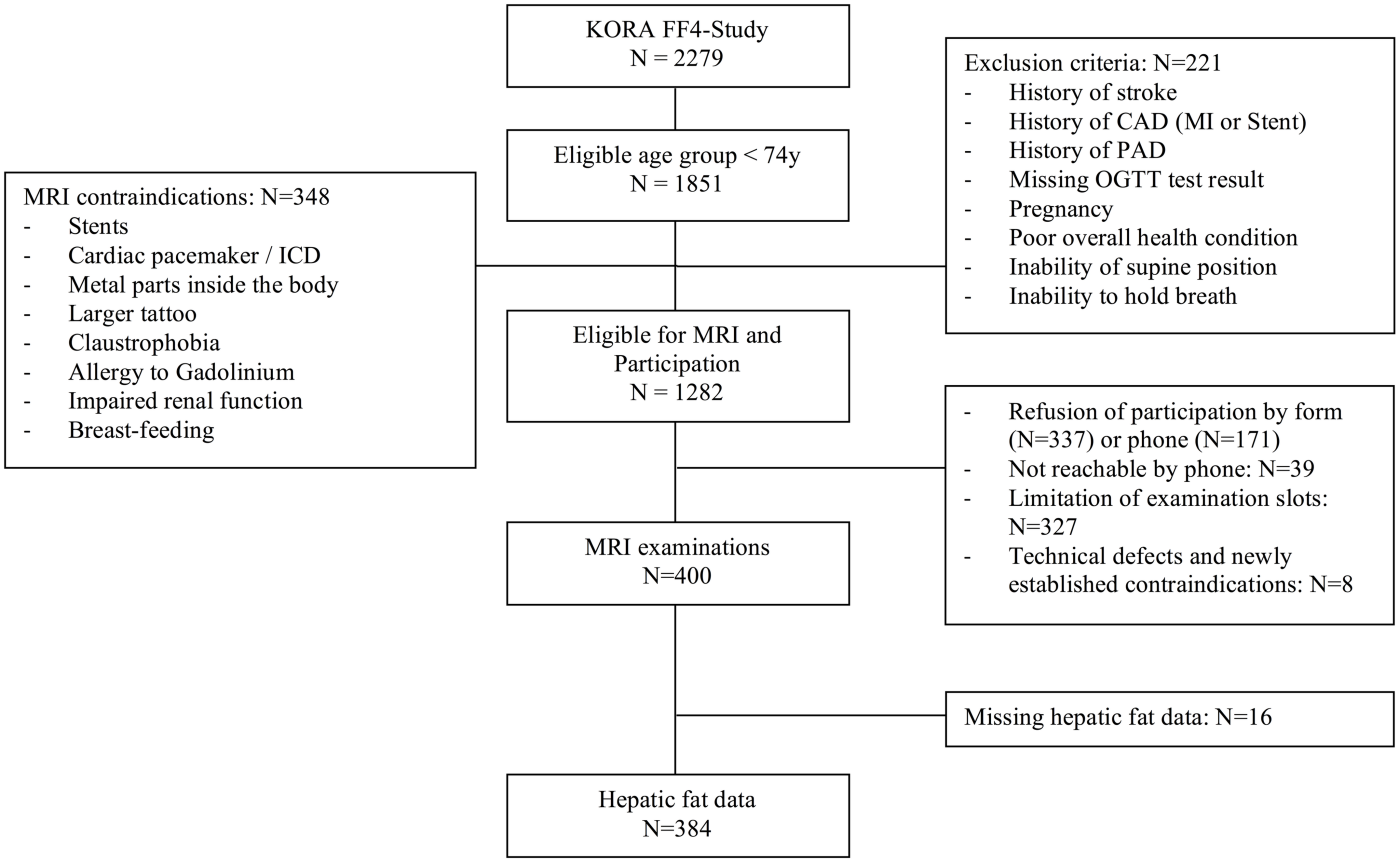
Participant flow diagram. *MRI* magnetic resonance imaging, *ICD* implantable cardioverter defibrillator, *CAD* coronary artery disease, *PAD* peripheral artery disease, *OGTT* oral glucose tolerance test, *KORA* Kooperative Gesundheitsforschung in der Region Augsburg.

Participants were included from the population-based cohort study Cooperative Health Research in the Region of Augsburg (KORA) consisting of German residents of the region aged 25 to 74 years at baseline examination. A total of 2279 of all 4261 individuals, who were part of the S4 baseline study (1999–2001), participated in the follow-up FF4 study conducted between 2013 and 2014. There were 1282 participants aged up to 72 years eligible for MRI examinations, 337 of these declined informed consent for the MRI study, 171 declined the telephone invitation, 39 were not reachable by telephone and 327 could not be included because of limited examination slots. Eight subjects could not be examined because of defective MR scanner or new contraindications. Hepatic fat data of 16 participants was missing, mainly because of software problems during acquisition and post-processing. This led to a sample of 384 individuals (223 men) aged 39 to 73 years. A flow chart containing the full exclusion criteria and MRI contraindications is shown as Fig 1. The complete study design, data collection and sampling method are also described in detail elsewhere [26].

The investigations were carried out in accordance with the Declaration of Helsinki, including written informed consent of all participants. All study methods were approved by the ethics committee of the Bavarian Chamber of Physicians, Munich (S4: EC No. 99186 and for genetic epidemiological questions 05004, F4 and FF4: EC No. 06068). The MRI examination protocol was further approved by the ethics committee of the Ludwig-Maximilian University Hospital, Munich.

### Assessment of smoking and alcohol consumption

KORA FF4 participants were asked about their alcohol consumption and smoking habits by a standardized interview.

Subjects were classified as current smokers if they smoked regularly (equal to or more than one cigarette per day) or irregularly (less than one cigarette per day) at the time of the interview, as former smokers, if they did not smoke at the time of the interview, but had smoked in the past and as never smokers if they never smoked in their lifetime.

Smoking was quantified as pack years, calculated by multiplying the number of packs of cigarettes smoked per day by the number of years the subject has smoked.

No alcohol consumption was defined as 0 g/day, moderate alcohol consumption as 0.1–39.9 g/day for men and 0.1–19.9 g/day for women and heavy alcohol consumption as ≥ 40 g/day for men and ≥ 20 g/day for women. These thresholds are based on the different metabolization of ethanol in men and women, mainly because of lower gastric alcohol dehydrogenase activity, which results in higher blood ethanol levels by the same amount of alcohol consumed [27] and on former studies regarding cardiovascular and overall-mortality [28–30].

### Covariates

All measurements were taken at the follow-up visit in the study center. For the definition of prediabetes and diabetes, we applied the WHO criteria [31]. OGTT was also performed in subjects without former diagnosis of diabetes or prediabetes. Body mass index (BMI) was calculated as weight divided by squared height (kg/m^2^) and waist circumference was measured in cm to the closest 0.1 cm at the smallest position between the lower rib and the upper margin of the iliac crest. Hypertension was defined as an increased systolic blood pressure (≥ 140mmHg) or increased diastolic blood pressure (≥ 90 mmHg), or the current treatment with antihypertensive medication. Measurements of laboratory parameters, such as triglycerides (TG), high-density lipoprotein (HDL), low-density lipoprotein (LDL), alanine transaminase (ALT), aspartate transaminase (AST), gamma-glutamyltransferase (GGT) and the calculation of Fatty Liver Index were described elsewhere [32, 33].

### Imaging protocol

All examinations were performed at a 3 Tesla Magnetom Skyra (Siemens AG, Healthcare Sector, Erlangen Germany) using an 18-channel body coil in combination with the table-mounted spine matrix coil. Subject position was supine. The overall examination time was approximately 60 minutes. All examinations were performed within three months after the visit at the study center. The study MR protocol included imaging of the brain, carotid arteries, heart, fat compartments and ectopic fat. All subjects underwent a liver imaging protocol that comprised a multi-echo Dixon sequence and multi-echo ^1^H-MRS. Details on the full and the liver imaging protocol are provided as supplementary material (see S1 Table).

#### Measurement of liver fat by MRI

**Fig 2:**
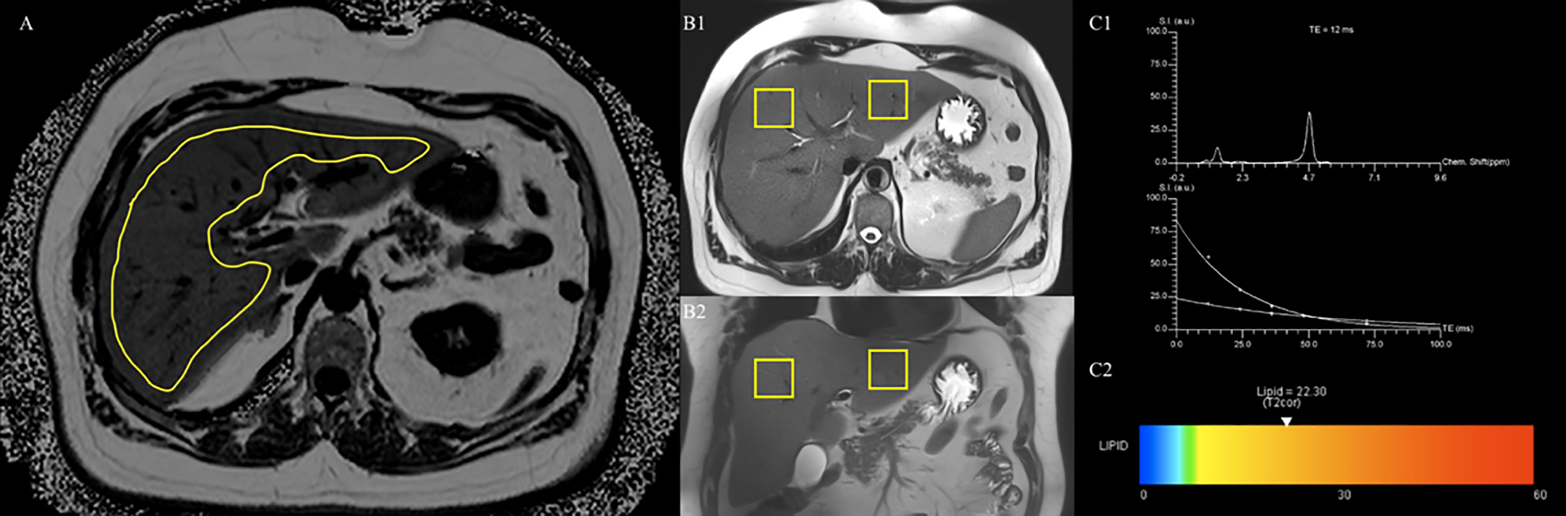
Example of multi-echo Dixon and multi-echo ^1^H-MRS. Image A shows an example of the multi-echo Dixon used for quantitative measurement of hepatic fat fraction (HFF) by placing the region of interest (yellow) in the liver parenchyma on the level of the portal vein avoiding large vessels. HFF measured by Dixon method in this subject was 28.1%. Images B show an example of the ^1^H-MRS method with voxels placed in the right (segment VIII) and left (segment II) liver lobe in axial (B1) and coronal (B2) slice. Results (C1, C2) are shown as a graph and a colored bar. The graph (C1) indicates the spectrum for the first acquired TE and the exponential decay fit for the five echoes and the colored bar (C2) presents the amount of liver fat. The average hepatic fat fraction measured by ^1^H-MRS was 27.9%.

MRI measurements were performed using a multi-echo Dixon approach with a volumetric interpolated breath-hold examination (VIBE) sequence with the following parameters: TR 8.90 ms, TEs opposed-phase of 1.23 ms, 3.69 ms, and 6.15 ms, TEs in-phase of 2.46 ms, 4.92 ms, and 7.38 ms, flip angle 4°, readout echo bandwidth 1080 Hz/pixel, matrix 256 × 256. Slice thickness was 4 mm. For the estimation of liver proton density fat fraction, confounding effects of T2* decay and the spectral complexity of fat were taken into account [34, 35]. Data were acquired during a single breath-hold of 15 seconds. By using OsiriX (Vers. 4.1 64-bit, Pixmeo SARL, Bernex, GE, Switzerland) the region of interest was manually drawn on one slice on height of the portal vein including the whole liver parenchyma avoiding large vessels and surrounding extrahepatic tissue as shown in Fig 2.

#### Measurement of liver fat by ^1^H-MRS and definition of FLD

A modified single-voxel spectroscopy sequence with stimulated-echo acquisition mode (STEAM), implementing the high-speed T2-corrected multi-echo (HISTO) technique was used for ^1^H Magnetic Resonance Spectroscopy (MRS), using the following parameters: TR 3000 ms, mixing time between second and third radiofrequency pulses 10 ms, and five TEs of 12.00 ms, 24.00 ms, 36.00 ms, 48.00 ms, and 72.00 ms, respectively. A total of 1024 points were acquired at a bandwidth of 1200 Hz, with one signal acquired by using a voxel size of 30 x 30 x 30 mm^3^. Voxels were placed in the right (segment VIII) and left (segment II) liver lobe. The sequence was acquired in a single breath-hold with an approximate duration of about 15 s. Spectrum post-processing and lipid content estimation were automatically performed. The principles have been previously described in detail [36]. Mean liver fat signal fraction was calculated from the measurement in the right and left liver lobe. An example of multi-echo ^*1*^*H-MRS* is provided in Fig 2.

The cut-off value for FLD was set to ≥5.56% (hepatic triglyceride level of 55.6 mg/g) corresponding to the 95^th^ percentile of the distribution of liver fat in 345 healthy subjects (non-obese, non-diabetic, minimal alcohol consumption), as defined in former studies [37, 38].

### Statistical analysis

Study sample characteristics were described separately for different smoking status groups as well as for different alcohol consumption groups by mean and standard deviation or numbers and percentages for continuous and categorical variables, respectively. Overall differences among exposure groups were assessed by one-way ANOVA or χ^2^-test.

Smoking status and alcohol consumption were separately associated with continuous HFF levels using linear regression models providing β-coefficients with 95% confidence intervals and with dichotomous FLD using logistic regression models providing odds ratios with 95% confidence intervals. Categories of smoking status and alcohol consumption were treated as dummy variables to estimate effects, reference being the respective non-exposed category. Results were presented unadjusted and adjusted for age, sex, BMI, hypertension, diabetes mellitus (DM) and alcohol consumption respectively smoking status as well as separately for the two outcomes of HFF measured by ^1^H-MRS and the Dixon method. In sensitivity analysis the association between cigarette smoking measured by pack-years and HFF was investigated. To demonstrate the association between continuous alcohol consumption measured as g/day and HFF, adjusted predicted values were plotted.

A p-value of <0.05 was considered as statistically significant, of <0.10 as borderline-significant. Analyses were performed using Stata 14.1 (Stata Corporation, College Station, TX, U.S.A.).

## Results

Details on the study population are provided in Table 1. Mean age of our study population was 56.2±9.1 years. Most participants were former smokers (168/384, 43.8%) or never smokers (139/384, 36.2%), a minority were current smokers (77/384, 20.0%). Furthermore, a majority were moderate drinkers (205/384, 53.3%) or non-drinkers (91/384, 23.7%) whereas fewer participants were heavy drinkers (88/384, 22.9%). Mean HFF was 8.8±8.0% measured by ^1^H-MRS and 8.5±8.4% measured by MRI. The highest measured HFF was 38.1% for ^1^H-MRS and 52.1% for MRI, the lowest HFF was 0.54% for ^1^H-MRS and 0.46% for MRI.

**Table 1:** Baseline characteristics.

	All	Non-Drinker	Moderate Drinker	Heavy Drinker	p-value	Never Smoker	Former Smoker	Current Smoker	p-value
	N = 384	N = 91	N = 205	N = 88		N = 139	N = 168	N = 77	
Age (years)	56.2 ± 9.1	55.6 ± 9.6	55.4 ± 9.0	58.5 ± 8.6	0.026	56.8 ± 9.3	56.5 ± 9.2	54.3 ± 8.6	0.129
Men	223 (58%)	37 (40.7%)	123 (60%)	63 (71.6%)	<0.001	74 (53.2%)	107 (63.7%)	42 (54.6%)	0.142
Smoking status					0.104				n/a
Never smoker	139 (36%)	41 (45.1%)	74 (36.1%)	24 (27.3%)		139 (100%)	n/a	n/a	
Former smoker	168 (44%)	31 (34.1%)	94 (45.9%)	43 (48.9%)		n/a	168 (100%)	n/a	
Current smoker	77 (20%)	19 (20.9%)	37 (18.1%)	21 (23.9%)		n/a	n/a	77 (100%)	
Cigarette smoking (pack-years)	12.0±17.7	12.0±17.1	9.9 ± 15.1	17.1 ± 22.3	0.007	0	16.4±18.7	24.9±18.4	<0.001
Alcohol consumption (g/day)	18.7 ± 24.0	0 ± 0	12.7 ± 10.1	52.2 ± 26.6	<0.001	14.7 ± 19.0	22.4 ± 27.7	17.9 ± 22.3	0.019
Body mass index (kg/m^2^)	28.1 ± 4.9	29.3 ± 5.5	27.9 ± 4.9	27.1 ± 4	0.011	27.4 ± 4.5	29.0 ± 5.2	27.1 ± 4.7	0.002
Waist circumference (cm)	98.5 ± 14.3	98.8 ± 15.4	98.4 ± 14.6	98.5 ± 12.6	0.967	96.5 ± 14	101.3 ± 14.8	96.2 ± 12.9	0.004
Systolic blood pressure (mmHg)	120.6 ± 16.9	120 ± 18.9	119.4 ± 15.4	124.2 ± 17.7	0.077	121.0 ± 16.0	121.5 ± 17.3	118.0 ± 17.3	0.310
Diastolic blood pressure (mmHg)	75.3 ± 10.0	75.1 ± 11.8	74.9 ± 9.4	76.3 ± 9.2	0.550	75.4 ± 9.6	75.8 ± 10.1	74.1 ± 10.4	0.490
Hypertension	132 (34%)	35 (38.5%)	60 (29.3%)	37 (42.1%)	0.069	44 (31.7%)	69 (41.1%)	19 (24.7%)	0.030
Diabetes mellitus	52 (14%)	16 (17.6%)	25 (12.2%)	11 (12.5%)	0.434	16 (11.5%)	28 (16.7%)	8 (10.4%)	0.280
HbA1c (%)	5.6 ± 0.7	5.6 ± 0.8	5.6 ± 0.8	5.5 ± 0.5	0.518	5.5 ± 0.9	5.6 ± 0.6	5.6 ± 0.5	0.812
Glucose (mg/dl)	104.2 ± 22.7	101.1 ± 20.0	104.3 ± 23.5	107.1 ± 23.4	0.214	103.4 ± 25.7	106.2 ± 22.8	101.2 ± 15.8	0.258
HDL-C (mg/dl)	61.9 ± 17.7	58.3 ± 16.7	61.5 ± 17.6	66.5 ± 18	0.007	65.4 ± 19.2	60.7 ± 16.6	58.1 ± 16	0.007
LDL-C (mg/dl)	139.6 ± 33.2	136.4 ± 31.1	141.3 ± 34.7	138.9 ± 31.7	0.497	138.1 ± 34.8	137.8 ± 30.9	146.1 ± 34.4	0.157
TG (mg/dl)	131.7 ± 85.3	116.4 ± 60.7	131.6 ± 82.5	148 ± 108.4	0.046	120.3 ± 77.5	141.6 ± 97.2	130.8 ± 67.5	0.092
ALT (μkat/l)	0.52 ± 0.30	0.48 ± 0.29	0.53 ± 0.32	0.53 ± 0.23	0.278	0.51 ± 0.32	0.55 ± 0.30	0.46 ± 0.23	0.076
AST (μkat/l)	0.42 ± 0.21	0.40 ± 0.16	0.43 ± 0.26	0.43 ± 0.14	0.514	0.41 ± 0.14	0.46 ± 0.28	0.37 ± 0.11	0.006
GGT (μkat/l)	0.67 ± 0.68	0.51 ± 0.44	0.62 ± 0.61	0.93 ± 0.92	<0.001	0.63 ± 0.60	0.70 ± 0.71	0.67 ± 0.72	0.654
Fatty Liver Index	54.5 ± 31.3	53.4 ± 32.1	53.9 ± 31.3	56.9 ± 30.8	0.705	49.1 ± 30.8	60.4 ± 31.2	51.2 ± 30.6	0.004
Hepatic fat fraction, 1H-MRS (%)	8.8 ± 8.0	7.8 ± 7.3	8.6 ± 7.9	10.3 ± 8.8	0.094	8.1 ± 7.7	10.5 ± 8.5	6.6 ± 6.6	<0.001
Hepatic fat fraction, MRI (%)	8.5 ± 8.4	7.5 ± 7.4	8.5 ± 8.6	9.4 ± 8.8	0.328	7.6 ± 8.1	10.2 ± 9	6.2 ± 6.6	<0.001
FLD, 1H-MRS (%)	198 (51.6%)	42 (46.2%)	101 (49.3%)	55 (62.5%)	0.057	68 (48.9%)	106 (63.1%)	24 (31.2%)	<0.001
FLD, MRI (%)	167 (43.5%)	37 (40.7%)	86 (42.0%)	44 (50.0%)	0.366	54 (38.9%)	92 (54.8%)	21 (27.3%)	<0.001
HFF≥15%, 1H-MRS (%)	72 (18.8%)	12 (13.2%)	38 (18.5%)	22 (25%)	0.128	20 (14.4%)	43 (25.6%)	9 (11.7%)	0.009
HFF≥15%, MRI (%)	71 (18.5%)	14 (15.4%)	37 (18.1%)	20 (22.7%)	0.437	20 (14.4%)	42 (25%)	9 (11.7%)	0.013

Data are given as mean **±** standard deviation or numbers and percentages. P-values are from one-way ANOVA or χ2-test. *HbA1c* hemoglobin A1c, *HDL-C* high-density-lipoprotein cholesterol, *LDL-C* low-density-lipoprotein cholesterol, *TG* triglycerides, ALT alanine transaminase, AST aspartate transaminase, GGT gamma-glutamyltransferase, *n/a* not applicable

### Association between smoking status and HFF

Former smokers had the highest and current smokers the lowest mean HFF rates (^1^H-MRS 10.5±8.5% vs. 6.6±6.6%; p<0.001 and MRI 10.2±9.0% vs. 6.2±6.6%; p<0.001, respectively). In unadjusted analysis former smokers showed increased HFF compared to never smokers (^1^H-MRS β = 2.37; p = 0.009 and MRI β = 2.64; p = 0.006). This association disappeared completely after multivariable adjustment. Interestingly, after adjusting for every single confounder separately, significance disappeared only after adjustment for BMI (^1^H-MRS β = 1.20; p = 0.142 and MRI β = 1.49; p = 0.088).

The prevalence of FLD was highest in former smokers and lowest in current smokers (^1^H-MRS 63.1% vs. 31.2%, p<0.001 and MRI 54.8% vs. 27.3%; p<0.001, respectively). In unadjusted analysis former smokers showed the highest odds for FLD (^1^H-MRS OR 1.79; p = 0.013 and MRI OR 1.91; p = 0.006, respectively) with never smokers as reference. After adjustment for age, sex, BMI, hypertension, DM and alcohol consumption this association disappeared (^1^H-MRS OR 1.07; p = 0.815 and MRI OR 1.25; p = 0.442), as shown in Table 2.

**Table 2:** Association of smoking status with hepatic fat fraction (HFF) and fatty liver disease (HFF≥5.56%).

Smoking status	HFF	unadjusted β (95% CI)	p-value	adjusted β* (95% CI)	p-value	FLD prevalence	unadjusted OR (95% CI)	p-value	adjusted OR* (95% CI)	p-value
	^**1**^**H**-**MRS**					^**1**^**H-MRS**				
Never smoker		Ref.		Ref.		48.9%	1		1	
Former smoker		2.37 (0.6; 4.14)	**0.009**	0.36 (-1.09; 1.81)	0.627	63.1%	1.79 (1.13; 2.82)	**0.013**	1.07 (0.59; 1.94)	0.815
Current smoker		-1.51 (-3.71; 0.69)	0.177	-1.13 (-2.9; 0.63)	0.208	31.2%	0.47 (0.26; 0.85)	**0.012**	0.37 (0.18; 0.76)	**0.007**
	**MRI**					**MRI**				
Never smoker		Ref.		Ref.		38.9%	1		1	
Former smoker		2.64 (0.78; 4.5)	**0.006**	0.71 (-0.87; 2.29)	0.374	54.8%	1.91 (1.21; 3.01)	**0.006**	1.25 (0.71; 2.22)	0.442
Current smoker		-1.39 (-3.7; 0.92)	0.237	-1.01 (-2.93; 0.91)	0.302	27.3%	0.59 (0.32; 1.08)	**0.088**	0.56 (0.27; 1.17)	0.124

*β*-coefficients are from linear regression, *OR* odds ratios are from logistic regression, *CI* confidence interval,

* adjusted for age, sex, BMI, hypertension, diabetes mellitus and alcohol consumption.

Significant values are shown in bold.

In our former smoker group BMI increased significantly with the amount of cigarettes consumed (β = 1.44; p = 0.007) with higher risk for obesity (as defined by BMI≥30kg/m^2^) (OR = 2.13; p = 0.005) compared to never smokers. Current smokers had a higher, but non-significant odds for obesity compared to never smokers (OR = 1.12; p = 0.746). All results are presented after adjustment for age, sex, hypertension, diabetes status and alcohol consumption, as shown in Table 3.

**Table 3:** Association of smoking status with BMI and the status of adiposity (BMI≥30 kg/m^2^).

Smoking status		unadjusted β (95% CI)	p-value	adjusted β* (95% CI)	p-value		unadjusted OR (95% CI)	p-value	adjusted OR* (95% CI)	p-value
	BMI					Adiposity				
Never smoker		Ref.		Ref.		23.0%	1		1	
Former smoker		1.61 (0.52;2.70)	**0.004**	1.44 (0.40;2.47)	**0.007**	38.7%	2.11 (1.28;3.49)	**0.004**	2.13 (1.25;3.62)	**0.005**
Current smoker		-0.32 (-1.68;1.03)	0.637	-0.03 (-1.31;1.24)	0.958	23.4%	1.02 (0.53;1.97)	0.953	1.12 (0.56;2.24)	0.746

*β*-coefficients are from linear regression, *OR* odds ratios are from logistic regression, *CI* confidence interval,

* adjusted for age, sex, hypertension, diabetes mellitus and alcohol consumption.

Significant values are shown in bold.

AST was found to be highest in former smokers and lowest in current smokers in comparison to all other subgroups (0.46±0.28μkat/l vs. 0.37±0.11μkat/l; p = 0.006). Fatty Liver Index was highest in former smokers and lowest in never smokers (60.4±31.2 vs. 49.1±30.8; p = 0.004). In the current smoker group a decreased odds for FLD was shown (^1^H-MRS OR 0.47; p = 0.012 and MRI OR 0.59; p = 0.088, respectively). The association remained after multivariable adjustment in ^1^H-MRS (OR 0.37; p = 0.007), but not in MRI (OR 0.56; p = 0.124). Results of the full analysis is shown in Table 2.

Among current smokers, HFF slightly increased with the amount of cigarettes consumed, but this association was not significant (^1^H-MRS β = 0.03; p = 0.456 and MRI β = 0.01; p = 0.742) after adjustment for all confounders.

### Association between alcohol consumption and HFF

Heavy drinkers showed the highest mean HFF rates compared to non-drinkers (^1^H-MRS 10.3±8.8% vs. 7.8±7.3%; p = 0.094 and MRI 9.4±8.8% vs. 7.5±7.4%; p = 0.328, respectively) and heavy drinking was positively associated with HFF compared to non-drinkers (^1^H-MRS β = 2.54; p = 0.034 and MRI β = 1.88; p = 0.136); the association was significant after multivariable adjustment (^1^H-MRS β = 2.99; p = 0.003 and MRI β = 2.49; p = 0.023). Furthermore, heavy drinkers had the highest prevalence (^1^H-MRS: 62.5% and MRI: 50.0%) and highest odds for FLD (^1^H-MRS OR 3.05; p = 0.008 and MRI OR 1.97; p = 0.095, respectively) after multivariable adjustment. They showed the highest levels of GGT, whereas non-drinkers revealed the lowest levels of GGT in comparison to all other subgroups (0.93±0.92μkat/l vs. 0.51±0.44μkat/l; p<0.001).

Moderate alcohol consumption was not associated with HFF in unadjusted analysis, but a borderline-significant positive association appeared after multivariable adjustment (^1^H-MRS: β = 1.54; p = 0.061 and MRI: β = 1.75; p = 0.050) with non-drinkers as reference. Odds for FLD were not significantly different compared to non-drinkers before adjustment (^1^H-MRS OR 1.13; p = 0.621 and MRI OR 1.05; p = 0.835). Complete data are shown in Table 4.

**Table 4:** Association of alcohol consumption with hepatic fat fraction (HFF) and fatty liver disease (HFF≥5.56%).

Alcohol consumption	HFF	unadjusted β (95% CI)	p-value	adjusted β* (95% CI)	p-value	FLD Prevalence	unadjusted OR (95% CI)	p-value	adjusted OR* (95% CI)	p-value
	**1H-MRS**					**1H-MRS**				
Non-drinker		Ref.		Ref.		46.2%	1		1	
Moderate drinker		0.86 (-1.12; 2.83)	0.395	1.54 (-0.07; 3.16)	0.061	49.3%	1.13 (0.69; 1.86)	0.621	1.46 (0.74; 2.89)	0.271
Heavy drinker		2.54 (0.19; 4.88)	0.034	2.99 (1.01; 4.96)	**0.003**	62.5%	1.94 (1.07; 3.53)	**0.029**	3.05 (1.33; 6.99)	**0.008**
	**MRI**					**MRI**				
Non-drinker		Ref.		Ref.		40.7%	1		1	
Moderate drinker		0.95 (-1.13; 3.03)	0.371	1.75 (0.00; 3.50)	0.050	42.0%	1.05 (0.64; 1.74)	0.835	1.36 (0.69; 2.67)	0.370
Heavy drinker		1.88 (-0.59; 4.34)	0.136	2.49 (0.35; 4.63)	**0.023**	50.0%	1.46 (0.81; 2.64)	0.210	1.97 (0.89; 4.36)	0.095

β-coefficients are from linear regression, *OR* odds ratios are from logistic regression, CI confidence interval

*adjusted for age, sex, BMI, hypertension, diabetes mellitus and smoking status.

Significant values are shown in bold.

**Fig 3:**
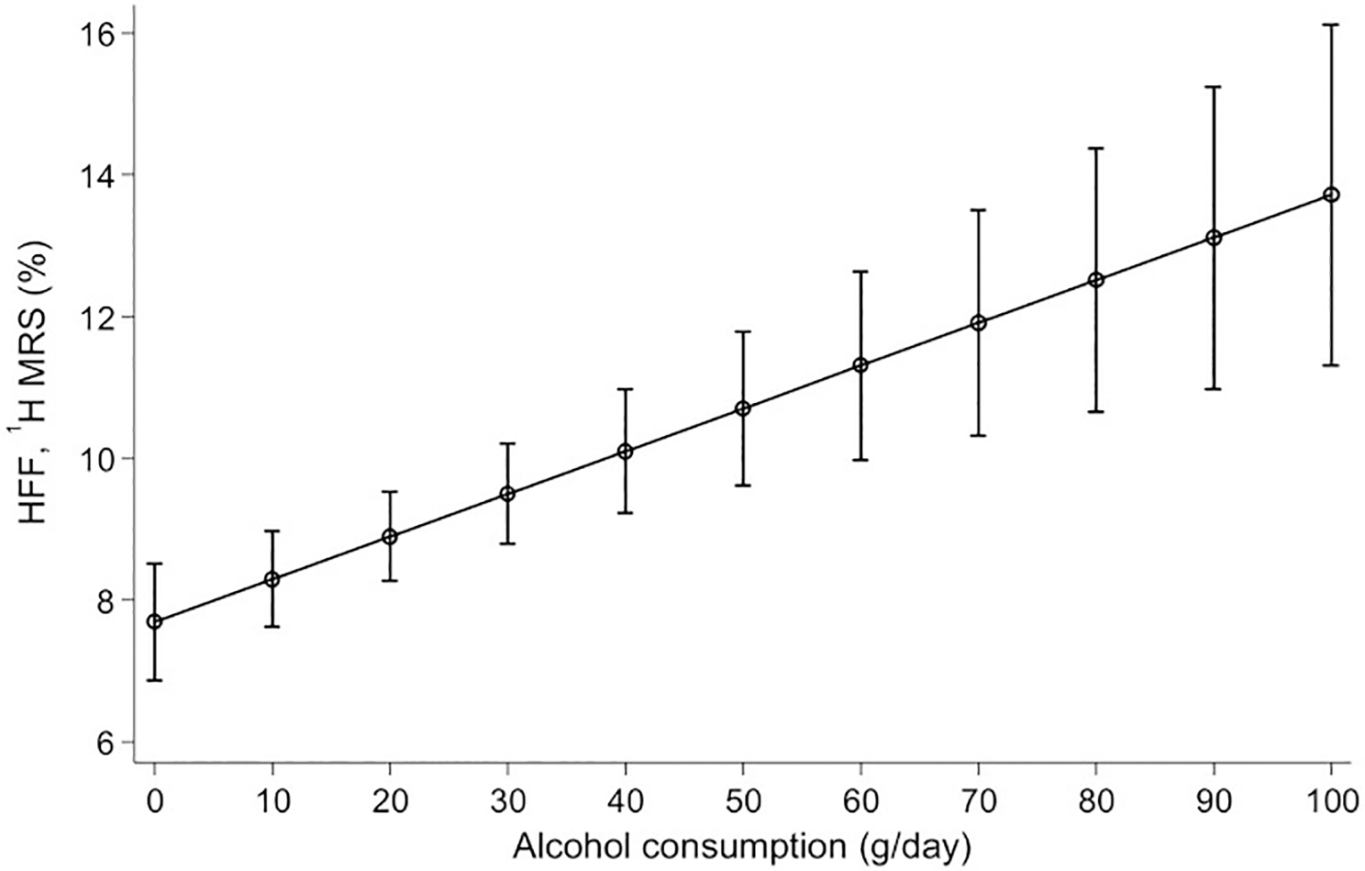
Adjusted linear prediction of hepatic fat fraction (HFF) via ^1^H-MRS according to alcohol consumption. Linear predictions with 95% confidence intervals of hepatic fat fraction measured by ^1^H-MRS according to alcohol consumption adjusted for age, sex, BMI, hypertension, diabetes mellitus and smoking status (p-value for β-coefficient <0.001).

**Fig 4:**
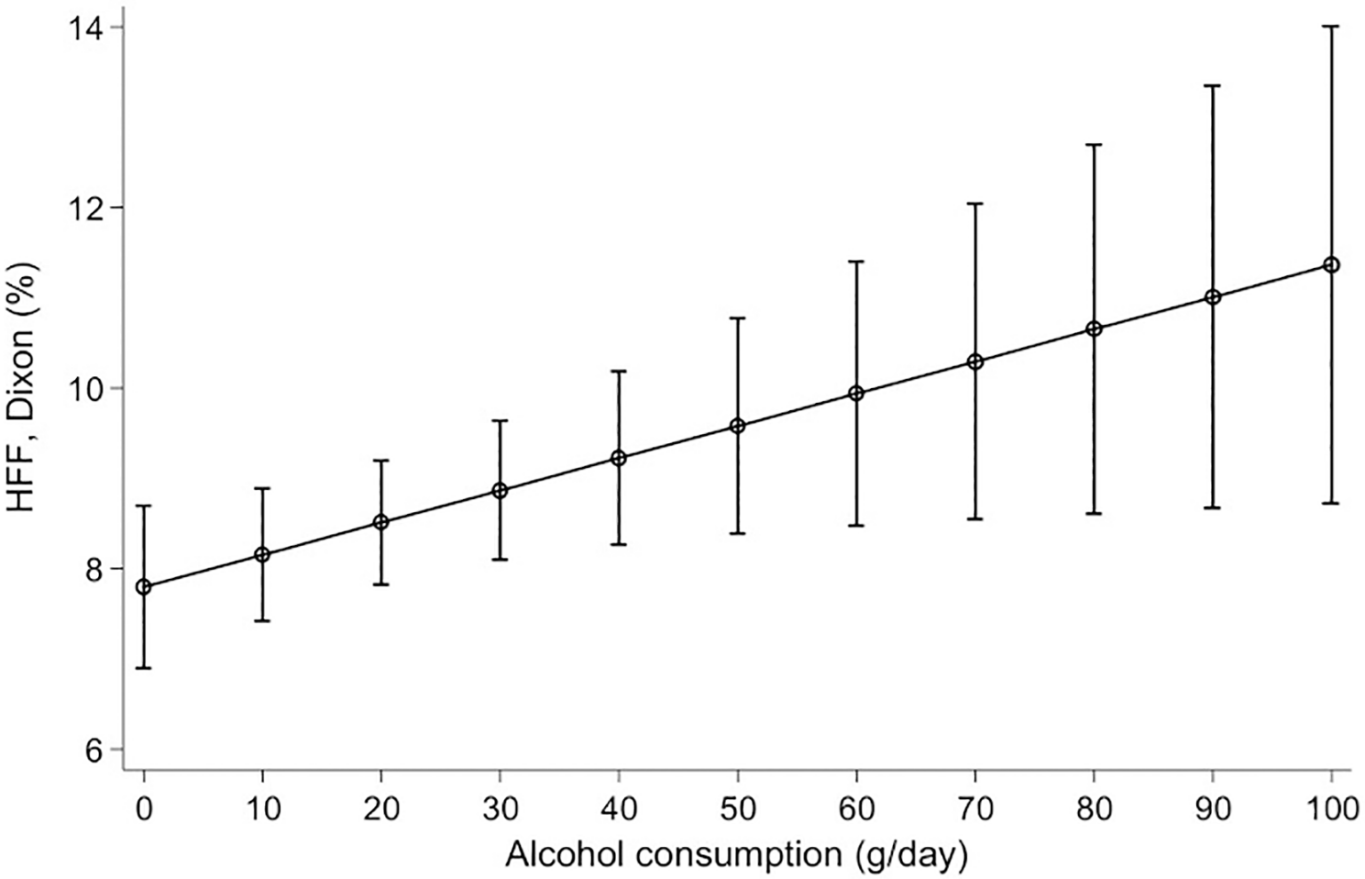
Adjusted linear prediction of hepatic fat fraction (HFF) via MRI according to alcohol consumption. Linear predictions with 95% confidence intervals of hepatic fat fraction measured by MRI according to alcohol consumption adjusted for age, sex, BMI, hypertension, diabetes mellitus and smoking status (p-value for β-coefficient = 0.026).

Adjusted linear predicted HFF according to alcohol consumption for ^1^H-MRS and MRI are shown in Figs 3 and 4, respectively.

## Discussion

In our study, current smokers showed the lowest FLD prevalence and had the lowest odds for prevalent FLD; however, HFF increased slightly among current smokers with the amount of cigarettes consumed. There was no significant difference in HFF when current smokers were compared to never smokers. Former smokers showed the highest rates of HFF and the highest odds for FLD; however, the association disappeared after adjustment for BMI.

Heavy alcohol consumption was positively associated with HFF and had higher odds for prevalent FLD compared to subjects who do not consume alcohol. Furthermore, heavy drinkers showed highest levels of ALT, AST, GGT and Fatty Liver Index compared to non- and moderate drinkers. The effect of moderate alcohol consumption revealed higher HFF in both MRI and ^1^H-MRS measurements with borderline-significance.

### Smoking habits and HFF

Our findings for the group of former smokers are partially in line with a study of Liu et al. [22] where former smoking was associated with higher risk for FLD compared to current light and moderate smokers. However, they found that heavy smokers had the highest risk of FLD, while our study found the highest odds for FLD in former smokers. This may in part be due to the fact that their measurements relied on ultrasonography measurements which are less precise than ^1^H-MRS and MRI [23, 24]. However, after multivariable adjustment of our results, the association of smoking and HFF disappeared for the group of former smokers. Further analysis revealed that BMI was the relevant confounder. Interestingly, former smokers showed increased BMI and highest odds for obesity in comparison to subjects who never smoke, which is often discussed in literature: Smokers tend to have a metabolically adverse fat distribution profile with more central obesity [39, 40] and lower BMI compared to non-smokers [12, 13] with increases in waist-to-hip ratio [14, 15]. Smoking cessation is often associated with weight gain [16, 17].

This weight gaining effect after smoking cessation may be based on hormonal mechanisms including a reduction of leptin and an increase of ghrelin, leading to an appetite-inducing effect [41, 42]. Furthermore, Hofstetter et al. showed that cigarette smoking increases the 24-hour energy expenditure by approximately 10 percent [43], which can be expected to decrease after smoking cessation and thus lead to weight gain. Insulin secretion may also play a role in this process. Stadler et al. demonstrated that former smokers had significant fasting hyperinsulinemia and fasting insulin resistance 3 months after cessation [44], which may further contribute to the weight gaining effect. Thus we suggest that there is no direct effect of smoking status on the deposition of fat in the liver in former smokers. Our results rather indicate that a higher BMI, a known risk factor FLD [45], mainly caused by the weight gaining effect after smoking cessation, may indirectly contribute to higher liver fat in former smokers.

Thus, former smokers should get early, reasonable attention in daily clinical routine in order to prevent relevant hepatic damage, which is underlined by the highest levels of AST and highest Fatty Liver Index compared to never and current smokers.

Current smokers had the lowest prevalence of and lowest odds for FLD in comparison to never smokers. In concordance with this, cigarette smoking is supposed to provide an appetite reducing effect, to expand the 24-hour energy consumption and to thus lead to lower BMI [12, 13, 42, 43].

Assuming similar mechanisms involved in the deposition of fat in the liver as in influencing body weight, mainly represented by BMI, our results are comparable to Dare et al., where current smokers had lower odds for obesity compared to never smokers with former smokers having highest odds compared to all other subgroups; among former and current smokers the risk for obesity increased with the amount of cigarettes consumed [18].

These findings underline the fact, that there are mechanisms associated with BMI, but also BMI-independent mechanisms involved in the deposition of fat in the liver by cigarette smoking.

### Alcohol consumption and HFF

In concordance to other studies, heavy drinking was associated with increased HFF and increased risk of FLD, which is based on well-established molecular mechanisms [3].

The role of moderate alcohol consumption is more controversially discussed. The findings of our study do not suggest a protective effect. Earlier studies suggested a protective effect of moderate alcohol consumption with regard to FLD [7–11], while others found negative effects including progressive fibrosis in subjects with FLD [46].

Some studies examined the association between liver fat and alcohol focusing on specific types of alcoholic beverages such as wine [7]. Red wine, for example, contains antioxidants, such as querceptin, which reduces liver oxidative damage [47] and may thus contribute to the inverse association between liver fat and wine consumption. Also the influence of certain lifestyle behaviors (i.e. activity, nutrition etc.) among wine drinkers compared to liqueur or beer drinkers may play a role in the findings of beverage type-specific studies. In our study all groups of drinkers were pooled together, no matter what sort of alcohol they mainly consume.

### Limitations and strength

There are certain limitations associated with our study. First, our study design is cross-sectional, therefore conclusions concerning temporality and causality of the relations are not possible. Second, we relied on self-reported smoking status and alcohol consumption, as assessed by a questionnaire. Third, our results are adjusted for all known confounders; however, there might be additional unrecognized effects.

One strength of our study lies in the accuracy of the HFF MRI and ^1^H-MRS measurement. In most previous studies that explored the association between cigarette smoking, alcohol consumption and liver fat, FLD was mainly diagnosed by serum liver enzymes, criteria for metabolic syndrome and ultrasonography (using indicative criteria like vascular blurring, deep attenuation and increased liver echotexture in comparison to liver-kidney contrast) [7–11, 19, 21, 22]. Ultrasound may be appropriate for detecting high hepatic fat accumulation, but is often confounded by severe fibrosis and not valuable in identifying mild steatosis [23, 24], which may lead to misdiagnosis. Multi-echo Dixon and ^1^H-MRS seem to be the most accurate modalities to detect hepatic steatosis, especially in mild disease with steatosis <30% [48–50]. In animal studies, these methods were shown to quantify the liver triglyceride content even more precisely than invasive histopathological methods [25]. Interestingly, results of MRI and ^1^H-MRS were essentially similar with regard to quantitative measurements and their association with smoking and alcohol consumption, underlining the equality of both approaches. Furthermore, examinations were performed in a relatively large population-based cohort with comprehensively assessed variables.

### Conclusion

Our results may help to further elucidate the complex interactions of smoking and alcohol consumption on liver metabolism. While our data may upfront suggest a protective effect of smoking, especially data on moderate alcohol consumption are conflicting and other detrimental effects of smoking and alcohol consumption on general health, e.g. the cardiovascular and pulmonary systems, are not taken into account. Furthermore, potential benefits of current smoking on FLD should be interpreted with caution, since the sample size of our current smokers group was lower compared to the other subgroups and a significant association with FLD was only detected in ^1^H-MRS but not MRI measurements. This is why further experimental, observational and interventional studies with a focus on poorly understood metabolic effects especially of smoking cessation and moderate alcohol consumption on the liver are needed to draw a final conclusion.

Moreover, our study underlines the necessity to use accurate, standardized, quantitative methods to study fatty liver disease. Some conflicting findings in comparison to former studies might be the result of different imaging entities. MRI and ^1^H-MRS today are fast and robust, quantitative methods and should be considered the preferred imaging modality for clinical studies if available.

## Supporting information

S1 Table Complete MR imaging protocol. Cardiovascular Whole-Body MRI Protocol: *TOF* Time of flight, *SWI* Susceptibility weighted imaging, *FLAIR* Fluid attenuated inversion recovery, *T2* T2 weighted, *SPACE* Sampling perfection with application optimized contrasts using different flip angle evolution, *T1w* T1 weighted, *T1w fs* T1 weighted fat saturated, *SAX* short axis, *LAX* long axis, *SSFP* Steady state with free precession, *MOLLI* modified look-locker inversion recovery, *LGE* Late gadolinium enhancement, *FLASH* fast low-angle shot, *VIBE* volume interpolated breathhold examination, *STEAM* Stimulated echo acquisition method, *HASTE* Half fourier acquisition single shot turbo spin echo, * voxel size.
(DOCX)
