# Supplementary material for: Alcohol consumption, but not smoking is associated with higher MR-derived liver fat in an asymptomatic study population
Source: PLoS One. 2018 Feb 5;13(2):e0192448. doi: 10.1371/journal.pone.0192448 (PMC5798849; doi:10.1371/journal.pone.0192448)
Supplement: S1 Table — Cardiovascular Whole-Body MRI Protocol: TOF Time of flight, SWI Susceptibility weighted imaging, FLAIR Fluid attenuated inversion recovery, T2 T2 weighted, SPACE Sampling perfection with application optimized contrasts using different flip angle evolution, T1w T1 weighted, T1w fs T1 weighted fat saturated, SAX short axis, LAX long axis, SSFP Steady state with free precession, MOLLI modified look-locker inversion recovery, LGE Late gadolinium enhancement, FLASH fast low-angle shot, VIBE volume interpolated breathhold examination, STEAM Stimulated echo acquisition method, HASTE Half fourier acquisition single shot turbo spin echo, * voxel size. (DOCX) [file pone.0192448.s001.docx]

**S1 Table. Complete MR Imaging Protocol.**

| **MR-Sequence** | **Weighting / Sequence Type** | **ST (mm)** | **Voxel size, In-plane (mm^2^)** | **FOV (mm)** | **Matrix** | **TR (ms)** | **TE (ms)** | **TI (ms)** | **Flip angle (°)** |
| --- | --- | --- | --- | --- | --- | --- | --- | --- | --- |
| **Brain** |  |  |  |  |  |  |  |  |  |
| TOF | TOF | 1 | 0.6 x 0.6 | 181 x 200 | 320 x 275 | 20 | 3.43 | N/A | 18 |
| SWI | SWI | 2.5 | 0.9 x 0.9 | 208 x 230 | 256 x 223 | 27 | 20 | N/A | 15 |
| FLAIR | T2, SPACE | 0.9 | 0.5 x 0.5 | 245 x 245 | 256 x 256 | 5000 | 389 | 1800 | 120 |
| **Plaque** |  |  |  |  |  |  |  |  |  |
| T1w carotid plaque | T1w fs | 3 | 0.3 x 0.3 | 165 x 220 | 320 x 240 | 800 | 13 | N/A | 180 |
| **Cardiac Function / Myocardium** | |  |  |  |  |  |  |  |  |
| Cine SAX | SSFP | 8 | 1.5 x 1.5 | 297 x 360 | 240 x 160 | 29.97 | 1.46, 10sl | N/A | 62 |
| Cine LAX | SSFP | 8 | 1.5 x 1.5 | 297 x 360 | 240 x 160 | 29.97 | 1.46 | N/A | 63 |
| MOLLI | T1w | 8 | 1.5 x 1.5 | 323 x 380 | 256 x 144 | 250 - 400 | 1.1 | 100 - 3500 | 35 |
| LGE | FLASH | 8 | 1.4 x 1.4 | 300 x 360 | 256 x 140 | 700 - 1000 | 1.55 | 280 - 345 | 20 - 55 |
| **Hepatic Adipose Content** |  |  |  |  |  |  |  |  |  |
| Dual-echo Dixon | VIBE | 3 | 1.2 x 1.2 | 308 x 380 | 320 x 195 | 4.10 | 1.23; 2.46 | N/A | 9 |
| Multi-echo Dixon | VIBE | 4 | 1.8 x 1.8 | 393 x 450 | 256 x 179 | 8.90 | 1.23; 2.46; 3.69; 4.92; 6.15; 7.38 | N/A | 4 |
| Spectroscopy | STEAM | N/A | 30 x 30 x 30* | N/A | N/A | 3000 | 12.00; 24.00; 36.00; 48.00; 72.00 | N/A | N/A |
| **Body Adiopose Content / Anatomy** | |  |  |  |  |  |  |  |  |
| Dual-echo Dixon | VIBE | 1.7 | 1.7 x 1.7 | 488 x 716 | 256 x 256 | 4.06 | 1.26; 2.49 | N/A | 9 |
| HASTE | T2 | 5 | 1.2 x 1.2 | 296 x 380 | 320 x 200 | 1000 | 91 | N/A | 131 |

**Cardiovascular Whole-Body MRI Protocol:** *TOF* Time of flight, *SWI* Susceptibility weighted imaging, *FLAIR* Fluid attenuated inversion recovery, *T2* T2 weighted, *SPACE* Sampling perfection with application optimized contrasts using different flip angle evolution, *T1w* T1 weighted, *T1w fs* T1 weighted fat saturated, *SAX* short axis, *LAX* long axis, *SSFP* Steady state with free precession, *MOLLI* modified look-locker inversion recovery, *LGE* Late gadolinium enhancement, *FLASH* fast low-angle shot, *VIBE* volume interpolated breathhold examination, *STEAM* Stimulated echo acquisition method, *HASTE* Half fourier acquisition single shot turbo spin echo, *** voxel size.
